# The Identification of the Uncertainty in Soil Strength Parameters Based on CPTu Measurements and Random Fields

**DOI:** 10.3390/s21165393

**Published:** 2021-08-10

**Authors:** Joanna Pieczyńska-Kozłowska, Irena Bagińska, Marek Kawa

**Affiliations:** Department of Civil Engineering, Wrocław University of Science and Technology, 50-370 Wrocław, Poland; irena.baginska@pwr.edu.pl (I.B.); marek.kawa@pwr.edu.pl (M.K.)

**Keywords:** geotechnical survey, soil strength parameters, CPT data, spatial variability

## Abstract

The present paper responds to the challenge of modeling uncertainty in soil strength parameters concerning its spatial variability in a situation of limited soil information. Understanding this uncertainty allows for the management of the risk of geotechnical structure failure. In the present work, this uncertainty is identified based on signals from the cone penetration test (CPT) device. Signals are directly transformed using existing interpretation methods (typically used as a source of mean values of parameters for a given range of depths) to obtain depth-varying effective strength parameters of the soil. The process is performed by incorporating data from two case studies from different locations in similar soil materials. First, Keswick clay from Australia, for which the results of both CPT and laboratory tests are available, is examined. Second, to further verify the obtained results, the soil from Poland called Świerzna clay, for which only CPT signals were available, is also tested. As shown, the variability of the transformed signals can be a good source of information for identifying uncertainty in soil strength. It agrees well with literature data and can be used to identify random fields describing soil parameters.

## 1. Introduction

The identification of model uncertainty using different types of measuring techniques is a subject of increasing interest [1,2], mainly because understanding the uncertainty in the model allows for the optimization of the management of specific resources. This problem also concerns the uncertainty in the values of soil strength parameters used in numerical models. Despite the constant development of in situ testing techniques for soils, the geotechnical recognition is still insufficient to determine soil properties at each point of the considered space. In typical numerical studies, constant values estimated based on a limited number of samples are used for characterizing specific soils. However, soil parameters are well-known to be subjected to strong spatial variability [3], which is one of the primary sources of uncertainty in the stability of geotechnical structures. Identification and modeling of this uncertainty allow for managing the risk of construction failure using so-called reliability-based design.

In recent decades, a method of modeling the spatial variability of soil parameters by stationary random fields (SRFs) has become popular. This is partially because random field theory (whose use in geotechnics was proposed by Vanmarcke [3]) has been relatively easy to incorporate into the finite element method [4,5]. In recent decades, the combined approach was used to analyze uncertainty in different types of geotechnical structures, including slopes [6], strip and rectangular foundations [7,8,9], and retaining walls [10]. Using this approach, the variability in soil properties can be accounted for within the framework of Monte Carlo simulations. The parameters to define a weak SRF consist of the probability distribution of the modeled property, estimated based on the measurement values, and the stationary autocorrelation function (ACF), which determines the correlation structure of the soil. The satisfactory modeling of structure uncertainty is often obtained even when the identification of the probability distribution of soil parameters is limited to its basic characteristics, i.e., the mean value and the coefficient of variation (COV) (c.f. [11]). However, the identification of ACF, or particularly its parameters, the so-called scales of fluctuations (SOFs), can have a great impact on the results [12]. The latter parameters are usually interpreted as the dimension of “clusters” where the field values are significantly correlated. Most of the existing studies show that the SRF modeling soil properties should be anisotropic, with the horizontal SOF being significantly greater than the vertical SOF, e.g., [6,7,8,9,13,14].

In most works dedicated to modeling soil material by SRF, the Mohr–Coulomb strength criterion is assumed. In such cases, shear strength parameters are usually the only properties modeled by random fields. These parameters are either the undrained shear strength (*s_u_*) [5], effective friction angle (*ϕ*′) [10], or both shear strength parameters, i.e., cohesion (c) and friction angle (*ϕ*′) (not always defined as the effective value) [4,9]. Sensitivity studies performed in a few of the mentioned works have shown that the variability of strength parameters has the greatest impact on the results for most cases where soil’s nonelastic behavior is considered. This is the case not only for the problem of the bearing capacity of shallow foundations (where it is intuitive) [7,15] or other problems related to the critical load but also for the deflection of the sheet pile wall supporting excavation in sands [10].

All data for the identification of the random field should be determined from measurements in the soil. The key to identifying the scale of fluctuations is the long series of data measured at a close interval. One of the best sources of such information is near-continuous surveys obtained using cone penetration test (CPT) devices [16,17]. However, the CPT device does not directly measure soil strength parameters but the values of other parameters related to soil strength, but not explicitly. For this reason, it is a common practice to assume that the fluctuation scales determined from the CPT measurements are equal to those for the strength parameters and to identify the mean and COV of the strength parameters using the results from laboratory tests that measure them directly [18,19]. These laboratory tests are time-consuming and expensive (especially in the case of their high quality) and, therefore, usually limited in number, which affects the accuracy of identification.

Moreover, since the fluctuation scales are identified for the stationary field, the trend must be subtracted from the obtained field data (measured directly by CPT). After generating the field of strength parameters, the trend for these values (if they exist) must be added, which raises additional problems with identifying and subtracting the trend from CPT measurements that is nontrivial (e.g., [17,20,21]) or determining the trend function for a limited number of laboratory tests.

As mentioned, the parameters measured by CPT and strength parameters are related. However, these relationships are not explicit. To reduce the uncertainty of field determined values, some researchers account for their simultaneous relations using a few different measurements, which can be statistically modeled using multivariate distributions. Such an approach can often be found in the works of Ching, Phoon, and their coworkers (e.g., [12,22]) and is often combined with Bayesian learning. Another idea is presented in work by Uzielli [16], where quantile regression is used to obtain a safe design value of *ϕ*′ based on CPT measurements. A review of some other approaches can be found in Cami et al. [23]. However, all of these approaches require a large database, including the results of field and laboratory tests performed on soil from nearby locations. As it seems to be a proper approach, these data are rarely available.

In the present work, a much simpler approach for identifying the SRF of strength parameters is proposed. The known methods for interpreting either undrained shear strength (*s_u_*) or effective shear strength parameters (*c*′ and *ϕ*′) from CPT measurements are used. However, while these methods are typically used to determine mean values for relatively large soil layers of the considered CPT profile, in the present work, they are used to transform the whole CPT signal (at each point where it was measured). Based on the transformed values, statistical information, i.e., mean values (or trends), coefficients of variation (COVs), and in the case of estimating both shear strength parameters, their cross-correlation coefficients (*ρ*) are investigated. The obtained values describing the uncertainty of soil strength parameters are then verified against laboratory tests from the literature. It is also checked whether the transformation formulas used for CPT measurements influence SOF values (i.e., whether the SOF determined for directly measured and transformed signals are different). All these procedures are performed to answer the following questions:

- Is it possible to identify stationary random fields only based on appropriately transformed CPT results?
- Do the variability and trends of selected strength parameters determined by CPT signal transformation behave similarly to the same values obtained from laboratory tests?
- Do any of the existing methods of estimation *c*′ and *ϕ*′ based on CPT provide reliable results for identifying random fields for these parameters, and do they also allow obtaining a reliable value for the cross-correlation coefficient between these fields?

From the presented results, based on surveys performed for the two cohesive soils, it appears that the proposed approach, although very simple, is promising. The obtained information is in good agreement with the literature data and the available laboratory tests. Thus, it seems that identification of random fields can be made directly from the transformed CPT signal in the absence of more accurate data. This information can be then further used in stochastic numerical analyses of geoengineering problems.

## 2. Materials and Methods

### 2.1. Method of Measurement

**Figure 1:**
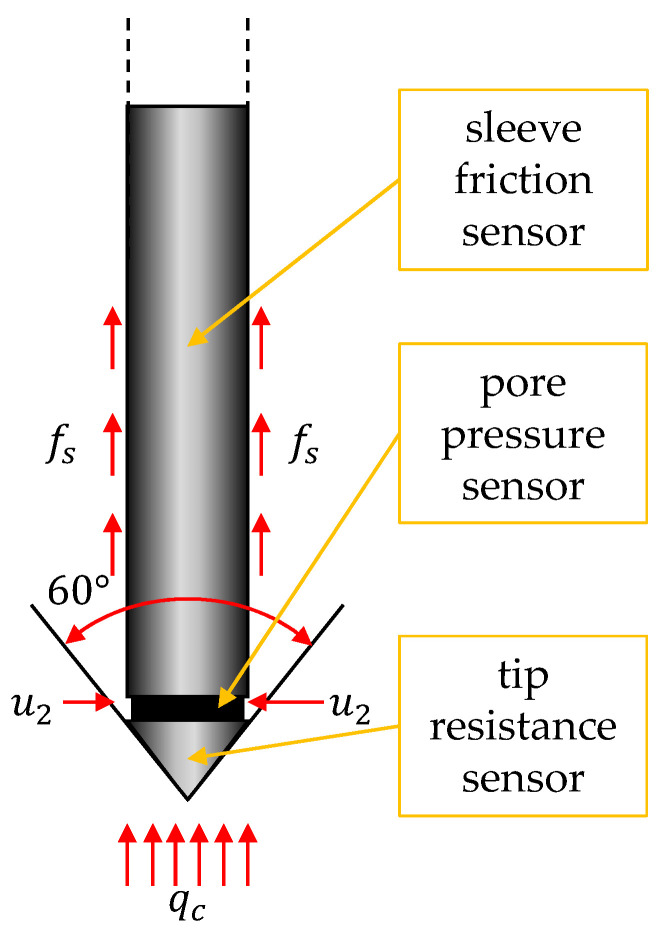
Schematic view of CPTu piezocone probe.

The cone penetration test (CPT) is a testing technique based on registering the resistances of a standardized probe (with a conic tip) when it is driven vertically into the soil at a constant speed. The technique started to be used in the 1930s in Denmark [24]. Initially, tests were performed using mechanical cones registering two parameters, i.e., cone tip resistance (*q_c_*) and sleeve friction (*f*_s_), along with the survey profile with an interval of tens of centimeters. This large interval is directly related to the design of mechanical cones [25]. Today, mechanical cones are used less and less, and the name CPT refers rather to the quasi-continuous surveys performed with electrical cones (with typical intervals of 0.5–2 cm). Within the EU, the guidelines on cone geometry (both electrical and mechanical) and test methodology are given in the standards [26,27]. Figure 1 shows a typical cone with a 10 cm^2^ base and 150 cm^2^ external surface at the tube shape. The cone tip diameter is between 35.7 and 36.1 mm. In addition to the above-standardized cone parameters, other geometries, sizes, and shapes of coneheads exist and are used, e.g., for special measurement conditions in organic soils.

Despite the standardized external geometry of the electric cone, its internal construction, i.e., the measurement and transmission system used, can vary considerably from one device to another. For example, inside the cones, measuring elements based on strain gauges or piezoelectric crystals and transmission systems may be based on electrical cables, radio waves, or even acoustic waves transmitted through the rods. The most commonly used electric cones are devices called piezocones characterized by the ability of quasi-continuous registration of three quantities, i.e., the already-mentioned cone tip resistance (*q_c_*), sleeve friction (*f_s_*), and also pore pressure at the shoulder (*u*). The test performed with such a cone is referred to as CPTU. Three possible locations for pore pressure filters have been developed over the years. One is with the filter placed in the middle of the tip height; the measurement is then referred to as *u*_1_. Second, the pore pressure filter is placed between the tip and the friction sleeve; the registration is then referred to as *u*_2_. If the filter is just above the friction sleeve, the measurement is referred to as *u*_3_. In research practice, it is very rare to use devices equipped with all three filter sensors. Due to their location, the *u*_1_ and *u*_3_ recorders are often damaged during the test. Thus, the most commonly manufactured and used electrical cone tips do not have any filter (register only *q_c_* and *f_s_* in the small interval) or are equipped with a *u*_2_ filter [28] (Figure 1).

With the development of electronics and the miniaturization of various measuring devices, various types of additional measuring equipment began to be attached to the basic piezocone devices [24,28]. Among others, modules equipped with geophones or accelerometers for downhole seismic measurements are used (SCPTU–seismic cone penetration test) [29,30]. It is also possible to attach a camera to locate chemical contaminants in the soil or verify the soil’s grain size (VisCPT–Vision Cone Penetrometer) [31], although it should be noted that this technique can be difficult to apply in cohesive soils. It is also popular to install additional temperature recording sensors or various electrical measurements to record the electrical conductivity or resistivity (RCPTU–resistivity piezocone penetration test). The information from this type of module is mainly useful in hydrated environments for identifying the different geological structures of the subsoil [32] or assessing possible liquefaction [33]. All these additional modules are usually applied locally to investigate the detailed features of the considered soil. Most of these tests do not affect the basic CPT registrations, although they may sometimes force an additional stop of the measuring column, which prolongs the test and will disturb the continuity of the measurement.

As the present paper focuses on the interpretation of basic CPT measurements and their application for the identification of uncertainties in soil strength parameters in both considered case studies (which will be described in more detail in the following subsection), the results obtained from the basic version of the electrical cone (without additional modules) were used. In both analyzed soil cases, the geometry of the cone was following the standard [27] (area of the base of the tip, 10 cm^2^, diameter of the tip and friction sleeve 35.7 mm, and area of friction sleeve 150 cm^2^). The differences between the cones used are of minor importance and should not affect the measured values. In the first considered case (Świerzna clay, see Section 2.4), the piezocone with *u*_2_ measurement, which measured all quantities every 2 cm, was used. In the second case (Keswick clay, see Section 2.4), the cone without filter (no pore pressure measurement), which registered *q_c_* and *f_s_* every 0.5 cm, was used.

### 2.2. Methods of Interpretation of Strength Parameters Based on CPT Data

To date, many relationships between CPT measurements and the strength parameters of soil have been developed. The most popular relation for undrained shear strength *s_u_* was defined by Lunne and Kleven [34] as:

(1) $$s_{u}=\frac{\left(q_{t}-\sigma_{vo}\right)}{N_{kt}},$$

where *σ_vo_* denotes the total overburden pressure at the elevation of the cone; $q_{t}=q_{c}+\left(1-a_{net}\right)u_{2}$ is the corrected total cone resistance ($a_{net}$ is a parameter associated with the geometry of the cone). Moreover, *N_kt_* is an empirical coefficient estimated between 14–16 [35]. In the present study, *N_kt_* was assumed to have a constant value of 15. Values of $\sigma_{v0}$ can be derived by integrating unit weight γ from surface to current depth, for example, using the formula by Bagińska [36].

(2) $$\gamma =11+2.4\cdot ln\left(f_{s}+0.7\right),$$

Two methods can be used to estimate the effective shear strength parameters (cohesion *c*′ and friction angle ϕ′) based on the CPT measurements. The first, much more popular method, described, e.g., in the papers by Senneset and Janbu [37], is often referred to as the NTH method. The other one, developed relatively recently, is based on solving two equations, as suggested in [38].

The NTH is based on the relationship for net cone resistance. According to Senneset and Janbu [37], in cohesive soils, where excess pore pressure can build up, the latter can be described as follows:

(3) $$q_{n}=q_{t}-\sigma_{v0}=N_{m}\left(\sigma_{v0}^{\prime }+a^{\prime }\right),$$

where *σ*′*_vo_* is the effective overburden pressure and *a*′ is the attraction parameter, which is related to *c*′ and *ϕ*′ as follows:

(4) $$a^{\prime }=c^{\prime }\cot\left(\phi^{\prime }\right),$$

and *N_m_* is cone resistance number defined as:

(5) $$N_{m}=\frac{N_{q}-1}{1+N_{u}B_{q}},$$

where *B_q_* = Δ*u/q_n_ (*Δ*u* = *u*_2_ − *u*_0_ denotes the excess pore pressure, where *u*_0_ is the pore pressure in drained conditions) and *N_u_* and *N_q_* can be calculated as:

(6) $$N_{u}=6\tan\phi^{\prime }\left(1+\tan\phi^{\prime }\right)$$

(7) $$N_{q}=K_{p}\exp\left[\left(\pi -2\beta \right)\tan\phi^{\prime }\right]$$

In the above formula, *K_p_* = (1 + sin (*ϕ*′))/(1 − sin (*ϕ*′)) is the passive lateral stress coefficient, and β is the angle of plastification (−40°<β<+30°), which defines the size of the failure zone after [34,37]. The value of β depends on the type of soil, its condition, and stress history.

Substituting Equations (5)–(7) into Equation (3), one obtains a direct relationship between *q_n_*, *a*′, tan (*ϕ*′), *B_q_,* and *β*. Values of $\sigma_{v0}$ and $\sigma_{v0}^{\prime }$ can be once again derived using the formula by Bagińska [36] (Equation (2)).

**Figure 2:**
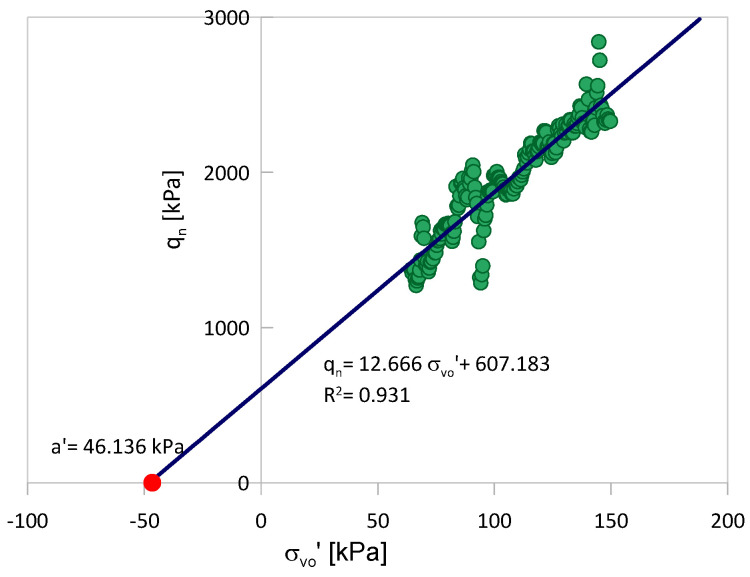
Determination of *a*′ based on the trend of *q_n_* against *σ*′*_vo_* based on results from a single CPT profile.

According to [37], the value of *a*′ can be determined using the trend in *q_n_* drawn against the effective overburden pressure *σ_vo_*′ (Figure 2). For a given *B_q_* and *β* and the determined value of *a*′, the mentioned relationship can be used to find *ϕ*′. Knowing *a*′ and *ϕ*′ *c*′ can also be obtained from Equation (4).

The other of the methods mentioned above, which is based on solving a system of equations (in this paper referred to as Method of Equations–MoE), assume that the effective cone resistance (*q_E_*) calculated as:

(8) $$q_{E}=q_{t}-u_{2}$$

can be estimated with the Terzaghi bearing capacity formula. The first equation is supplemented by the second equation describing the frictional behavior of soil on the sleeve of the CPT device. The complete system can be written as follows:

(9) $$\{\begin{array}{c} c^{\prime }N_{c}+\sigma_{v0}^{\prime }N_{q}+0.5\gamma BN_{\gamma }=q_{E}=q_{t}-u_{2}\\ c^{\prime }+\sigma_{hc}^{\prime }\tan\delta =f_{s} \end{array}$$

Concerning deep bearing capacity factors by Senneset and Janbu [37] and the analytical failure model shown by Eslami and Fellenius [39], factors in Equation (9) are proposed to be calculated as follows:

(10) $$N_{q}={\left(\tan\phi^{\prime }+\sqrt{1+\tan^{2}\phi^{\prime }}\right)}^{2}\exp\left(2\xi \tan\phi^{\prime }\right),$$

(11) $$N_{c}=\frac{\left(N_{q}-1\right)}{\tan\phi^{\prime }}$$

(12) $$N_{\gamma }=2\left(N_{q}+1\right)\tan\phi^{\prime },$$

In the above formulas, ξ denotes the angle defining the shape of the shear surface around the tip of the cone. The angle ranges from $\frac{\pi }{3}$ for soft clays to 0.58π for dense sands. In the present study, the following formula was assumed after [38] $\xi =3.05\cdot 10^{-3}\left(\frac{q_{t}}{p_{a}}\right)+1.2$ (*p_a_* is the atmospheric pressure). Additionally, *B* is equal to the cone diameter, and δ denotes the friction angle between the soil and the penetrometer sleeve, which was assumed to be 2/3 of the friction angle. The values of *γ* and $\sigma_{v0}^{\prime }$ can be obtained from Equation (2) (the latter by integration as mentioned above). Finally, the effective horizontal stress perpendicular to the penetrometer body $\sigma_{hc}^{\prime }$ (Equation (9)) acting on the sleeve according to [40] can be calculated as:

(13) $$\frac{\sigma_{hc}^{\prime }}{\sigma_{h0}^{\prime }}=7.89\cdot 10^{-4}{\left[\frac{q_{t}-\sigma_{h_{mean}}}{\sigma_{h_{mean}}^{\prime }}\right]}^{1.44}$$

where $\sigma_{h0}^{\prime }$ is the horizontal effective overburden stress calculated as *σ_ho_*′ = *k*_0_*σ_vo_*′ = (1 − sin*ϕ*′)*σ_vo_*′ and $\sigma_{h_{mean}}$ and $\sigma_{h_{mean}}^{\prime }$ denote the mean total and effective stresses, respectively.

As presented in the works of many researchers (e.g., [41]), the NTH method exhibits a good correlation with the results of laboratory tests. Similar conclusions can be drawn from the work by Motaghedi and Armaghani [38].

Typically, these methods are used to find some mean value of soil strength parameters for a selected layer (given range of depth). In the following section, all three presented methods (the method for calculating *s_u_* as well as two methods for calculating *c*′ and *ϕ*′) are applied to all points of the CPT profiles obtained from the two considered case studies to assess both the point and spatial variability in the strength parameters of cohesive soils.

### 2.3. Method for Identification of Vertical SOF

As mentioned in the introduction section, an important part of identifying the uncertainty of soil strength parameters in modeling them using a random field is identifying the scale of fluctuation. A typical method of determining the SOF based on CPT results [17,23] includes fitting the assumed theoretical ACF (with the SOF being the fitting parameter) to the so-called experimental ACF. The values of the latter are calculated for subsequent values of the lag distance between observations in CPT profile (multiplication of observation interval Δ*τ*) *τj* = *j*Δ*τ*, *jεN*, as [24]:

(14) $$\hat{\rho }\left(\tau_{j}\right)=\frac{1}{\nu^{2}k}\overset{k-j}{\underset{i=1}{\sum }}\left(X_{i}-\mu \right)\left(X_{i+j}-\mu \right)$$

where $X_{i}$ is the single observation of the parameter for which the scale is determined, *k* is the number of observations in the sample, and *μ* and *ν*^2^ denote the mean value and variance of the observations. According to the method [17,20,23], before applying Equation (14), all observations need to be initially detrended and normalized (trends and mean values are determined separately for each CPT).

For the theoretical ACF, the exponential Markov model is probably the most popular in geotechnics

(15) $$\rho \left(\tau \right)=\exp\left\{\frac{-2\left|\tau \right|}{\theta }\right\}$$

where θ denotes SOF value. 

This method was also used in the present paper to find the value of θ for both sensing data and transformed/interpreted signals. In all cases, the Markov model was assumed as the experimental ACF. The obtained values are also presented in the following section.

### 2.4. Materials–Sensing Data

The examined material is overconsolidated clay. The analysis is based on CPT measurements obtained in two independent case studies. The first considered example is clay from Świerzna, southwestern Poland. Characteristics for that soil (including OCR and plasticity index (IP) are collected in Table 1. In this location, a CPT survey consisting of nine CPTs located in a close-to-regular grid with a spacing of approximately 3 m in both directions was performed. Some results of that survey were previously presented in [41,42]. No free groundwater table was observed during the drilling and CPT measurements in the profile. The *u*_2_ registrations locally reached values up to several tens of kPa. Due to the lack of a free groundwater table in further calculations, the value of *u*_0_ was assumed to be zero.

As the second example, a case study performed by Jaksa [19,43] in Keswick clay, Adelaide, South Australia, was selected. The main reason for using the results by Jaksa was the availability of laboratory test results on the undrained shear strength *s_u_* carried out for the same soil, which could be used to verify the results obtained in the present study. The results of these tests will be presented in the next subsection. The basic characteristics for the Keswick clay are again collected in Table 1.

The original study by Jaksa [43] included over 100 CPT readings with a depth of app. 5 m (with the Keswick clay layer located approximately 2.2–2.6 m below the surface). The authors of the present paper analyzed approximately 40 test results from Jaksa’s database. In most analyzed cases, the *q_c_* registrations for the studied layer indicated a decrease in the values with depth (which is not typical and can be associated with a changing plasticity state of soil). For the present study, five test results from Jaksa [43] characterized by linear increases in *q_c_* and *f_s_* values (similar to the case of clay from Świerzna) were selected. Using the notation by Jaksa [19] tests A0, B0, J9, J10, and K9 were chosen. In some other tests, strongly variable readings (even for the range of depths that should correspond to the layer of Keswick clay) were observed. According to [42], none of the performed CPTs encountered the groundwater table; therefore, in this case, *u*_0_ = 0 was assumed for further calculations.

In the first step of analyzing sensing data from Świerzna, the homogeneous layer of cohesive soil was selected based on values of the nonnormalized soil behavior type index (*I_SBT_*) following the classification given in [44]. The *I_SBT_* parameter is defined as follows:

(16) $$I_{SBT}=\sqrt{{\left[3.47-\log\left(\frac{q_{c}}{p_{a}}\right)\right]}^{2}+{\left(\log\left(\frac{100\;f_{s}}{q_{c}}\right)+1.22\right)}^{2}}$$

where *p_a_* denotes atmospheric pressure (equal to 100 kPa).

**Figure 3:**
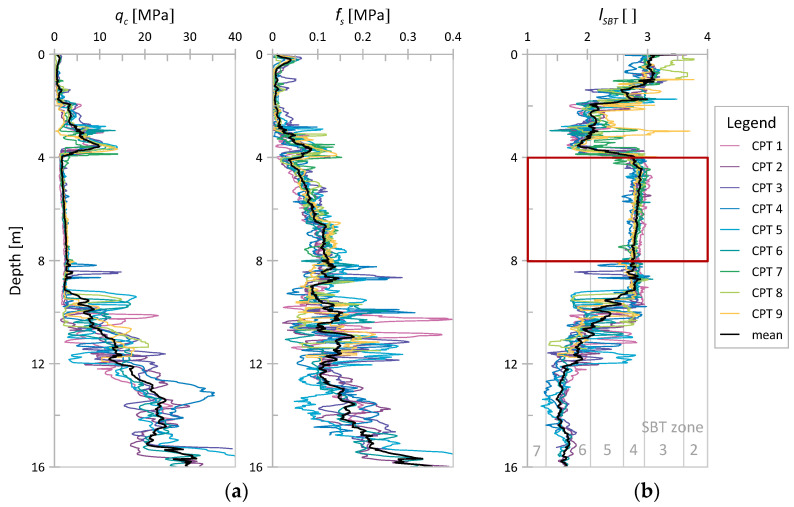
Results of nine CPTs from Świerzna [42]; (**a**) cone resistance *q_c_* and sleeve friction *f_s_* vs. depth; (**b**) nonnormalized SBT index (*I_SBT_*) vs. depth.

**Figure 4:**
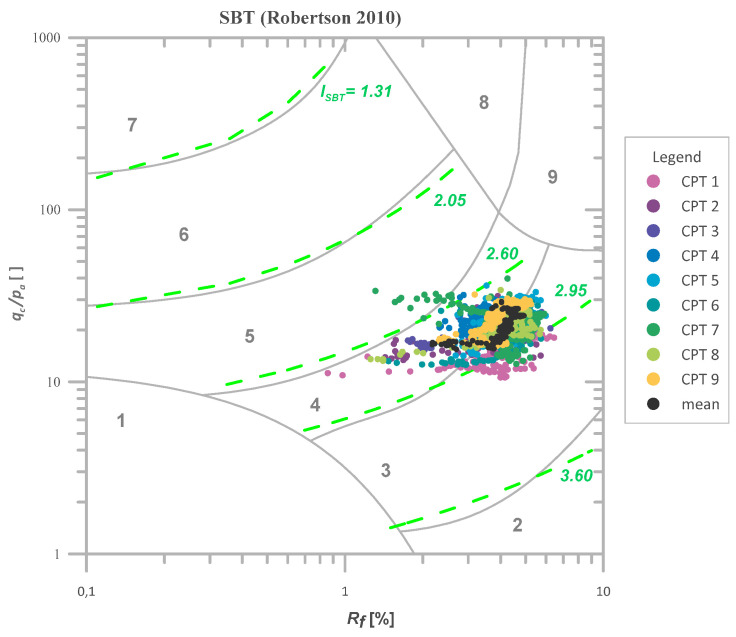
The values of ISBT in the selected homogeneous layer (4–8 m below the surface) for CPTs from Świerzna [42] presented in Robertson’s classification [44].

In Figure 3, the results for *q_c_* and *f_s_* and I_SBT_ values for Świerzna clay are presented. A homogeneous layer of cohesive soil can be observed between 4.0 and 8.0 m below the surface. For that layer, *q_c_* values ranged from 1.06 to 4.0 MPa with the mean value of 2.15 MPa, and *f_s_* values ranged from 0.01 to 0.163 MPa with the mean value of 0.09 MPa The I_SBT_ values obtained for that material are once again presented in Figure 4 in Robertson’s classification [44]. As seen, the material is indeed very homogeneous. The soil data shown in Figure 3 are mostly classified as SBT 4 (silty clay to clay). Based on the granulometric analysis performed independently, the soil was recognized as clay with the addition of sand.

**Figure 5:**
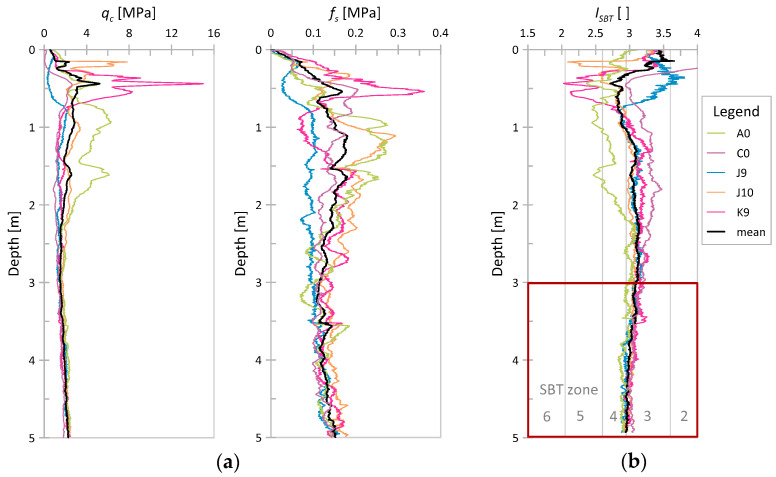
Results of five CPTs performed in Keswick clay [43]; (**a**) cone resistance *q_c_* and sleeve friction *f_s_* vs. depth; (**b**) nonnormalized SBT index (*I_SBT_*) vs. depth.

**Figure 6:**
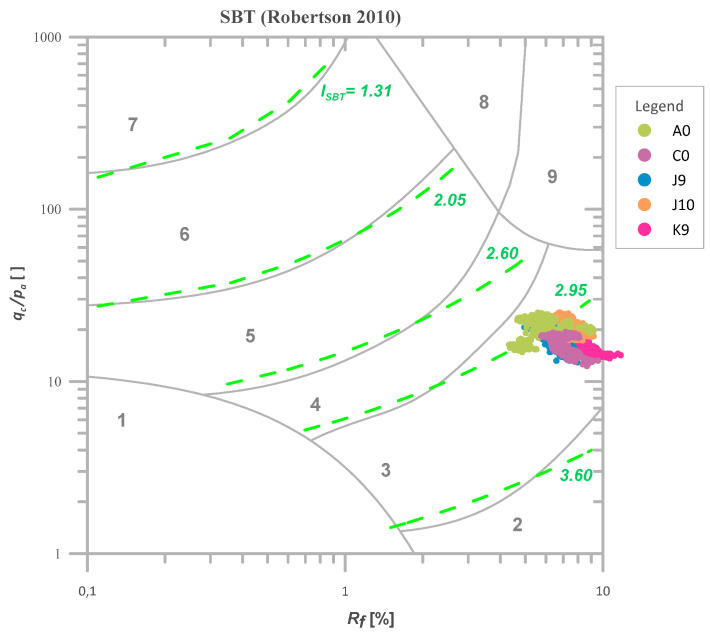
The values of I_SBT_ for the selected homogeneous layer (3.0–5.0 m below the surface) for CPTs performed in Keswick clay [43] presented in the Robertson’s classification [44].

Using a similar procedure for the case of Keswick clay, a homogeneous layer between 3.0 and 5.0 m below the surface was found and selected for further analysis. The results of *q_c_*, *f_s_*, and *I_SBT_* for the considered tests are shown in Figure 5. This time the *q_c_* values ranged from 1.23 to 2.51 MPa with the mean value of 190 MPa, and *f_s_* values ranged from 0.07 to 0.19 MPa with the mean value of 0.13 MPa. Figure 6 presents the results from the selected layer in Robertson’s classification [44].

### 2.5. Materials–Verification Data

Previously mentioned laboratory tests for strength parameters of Keswick clay (which can be used as reference values for identification of the uncertainty of strength parameters for that soil) are mainly *s_u_* tests consisting of several triaxial UU (unconsolidated undrained) and CU (consolidated undrained) tests carried out on samples taken at depths up to over a dozen meters. These results can be found in Appendix C of the PhD thesis by Jaksa [43]. For the present study, values representing the test from depths up to 10 m (a total of 87 UU and CU) were used as reference values.

**Figure 7:**
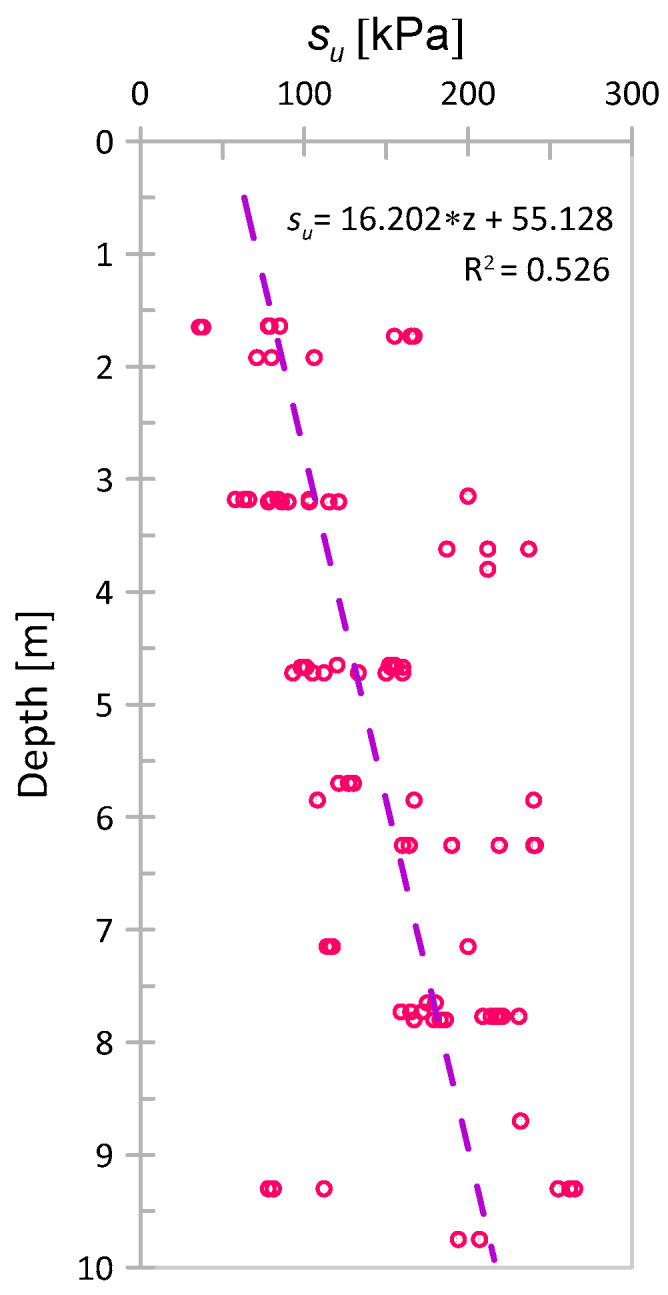
Results of *s_u_* for Keswick clay with depth based on Appendix C of the PhD thesis by [43] together with the obtained linear trend.

These laboratory tests show a clear increasing trend with depth. The linear trend equation obtained from these results and COV values calculated for three different series of samples (taken at depths of approximately 3.2, 4.7, and 7.7 m) is summarized in Table 2. Mean values used to calculate those COVs were taken as the value of the trend function for the respective depth. Additionally, in Figure 7, the considered results for *s_u_* with depth are presented together with the determined linear trend. In the following part of the work, we will compare the trend obtained from interpreted CPT measurements with these data.

In the case of both considered locations, i.e., Świerzna and Keswick, the results of laboratory tests concerning *c*′ and *ϕ*′ were not available (or their number was not sufficient to verify their variability). Therefore, it was decided that the verification of variability measures of *c*′ and *ϕ*′ would be based on literature data obtained in cases where a satisfactory number of laboratory tests were performed for similar soils. Several studies are investigating the variability in the effective shear strength parameters of soils. Values of COV and cross-correlation coefficient *ρ* for many different cohesive soils were presented, e.g., in a paper by Greco [45]. Table 3 shows some typical values from this work and some other works obtained in triaxial and direct shear tests. It appears that despite the test type, the COV for effective cohesion *c*′ is usually much higher (three times or more) than the COV for friction angle *ϕ*′.

Additionally, based on the analysis of both direct shear and triaxial tests of cohesive soils, it appears that in the case of *c*′ and *ϕ*′, very weak trends (if any) of these values with depth are observed (e.g., [46]). Thus, the COV and correlation coefficient ρ form probably the satisfactory set of measures of point variability (and cross-correlation) for these parameters. In further analysis, the data shown in Table 3 will be used as reference values.

## 3. Results

In this section, the results of the transformation of sensing data presented in Section 2.4 obtained for every point of the considered layers using the interpretation methods presented in Section 2.2 are shown. Based on the transformed data, the variability of shear strength parameters of the considered soil is investigated. Additionally, the vertical SOF for the original and transformed signal and cross-correlation coefficients for *c*′ and *ϕ*′ are identified.

### 3.1. Determined Values of Undrained Shear Strength s_u_

**Figure 8:**
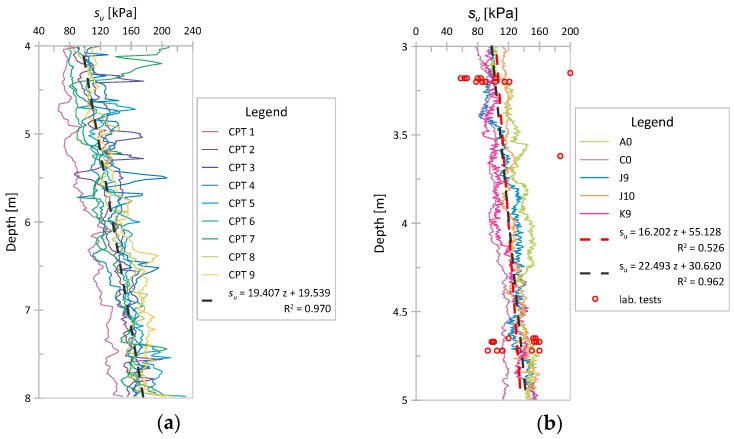
The *s_u_* values base on CPT vs. depth. Mean value marked with the black line; (**a**) CPTs for Świerzna clay; (**b**) CPTs for Keswick clay.

Based on *q_c_* measurements obtained from both analyzed case studies, the undrained shear strength (*s_u_*) was determined using Equation (1). In both cases, only the results for selected geotechnical layers were used. The *N_kt_* parameter was assumed to be equal to 15. The total vertical overburden pressure values were obtained using the varying unit weight of the soil calculated based on Equation (2). Figure 8 shows the variability in the *s_u_* value with depth. In both cases, a strong increasing trend of *s_u_* with depth can be observed. In Figure 8b, values derived from CPT and their trend and the results of laboratory tests of *s_u_* with their trend are shown (the R^2^ coefficients for both trends displayed are given in the legend section of the graph). As seen, the two trends (from in situ and laboratory tests) derived for a given layer are practically identical (although their coefficients are different).

**Figure 9:**
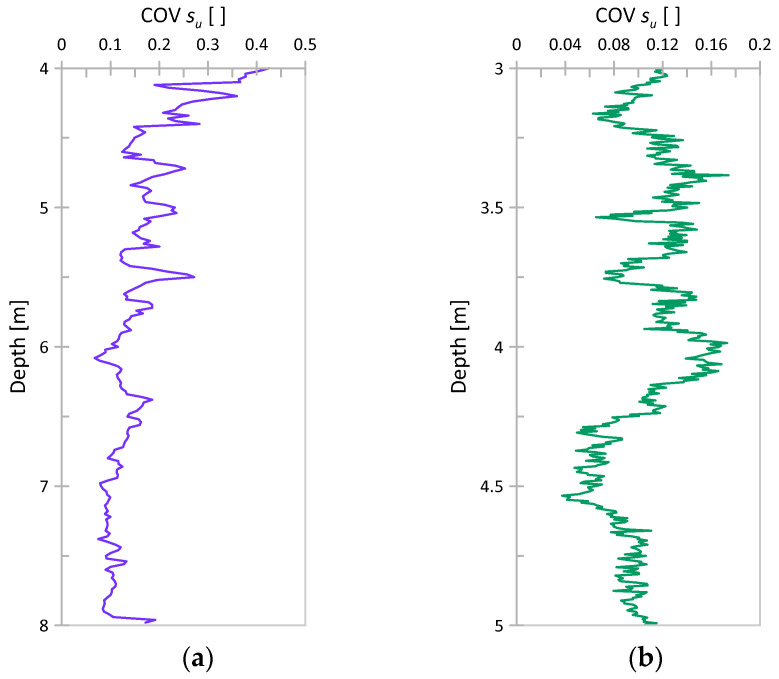
The COV of *s_u_* (based on CPT) vs. depth (**a**) CPTs for Świerzna clay (**b**) CPTs for Keswick clay.

From the results obtained, COVs were also determined. The standard deviation value obtained at a given depth was divided by the value of the trend function at that depth. Diagrams of COV versus depth are presented for both considered cases in Figure 9. As seen, the COV plots exhibit a weak decreasing trend with depth, which is more visible in the case of Świerzna, where a thicker layer of soil is analyzed. However, it seems that this trend at some point becomes negligible. The range of COV values obtained for the considered range of depths as well as the mean COV values are summarized in Table 4.

### 3.2. Shear Strength Parameters ϕ′ and c′ Determined by the NTH Method

Using the same values of CPTu measurements, the shear strength parameters *c*′ and *ϕ*′ were determined using the NTH method. The values of *a*′ were determined separately for each profile to consider the variability in parameter *a*′ (and thus variability *c*′). An example of such a determination based on the trend obtained in the *q_n_-σ_vo_′* space for a single profile section is presented in Figure 2 in the previous section. All obtained values of *a*′ are collected in Table 5. For comparison, the values obtained based on the global trend (values from all CPT profile sections associated with the considered layer) are also shown in the table.

All other steps of the NTH method were performed in the same way as described in Section 2.2. Using *a*′ value specified for each profile *ϕ*′ was calculated based on Equation (3) after substituting Equations (5)–(7) and assuming *B_q_* = 0 (due to very low or unknown pore pressure values) and *β* = 0. Finally, using the obtained *ϕ*′ and *a*′, *c*′ values were determined (also for each point of the profile) according to Equation (3).

**Figure 10:**
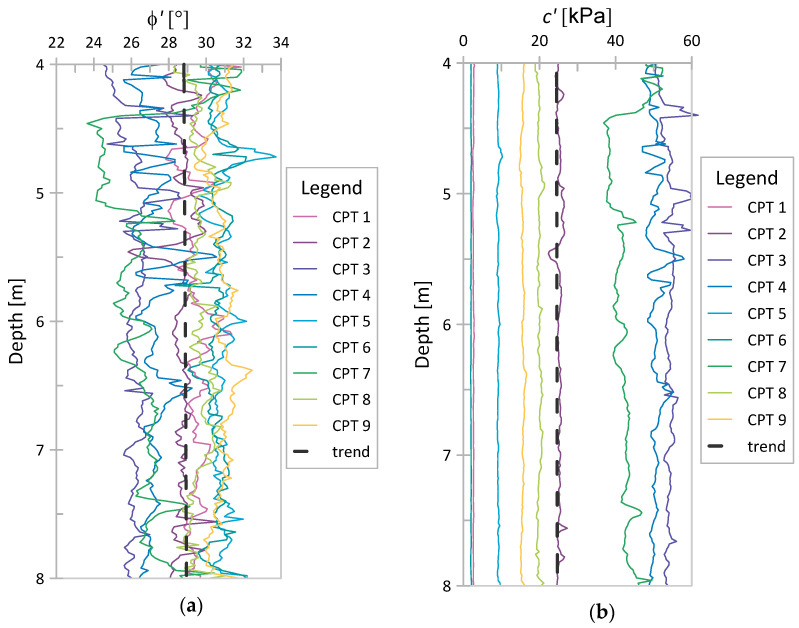
NTH results for Świerzna clay: (**a**) *ϕ*′; (**b**) *c*′.

**Figure 11:**
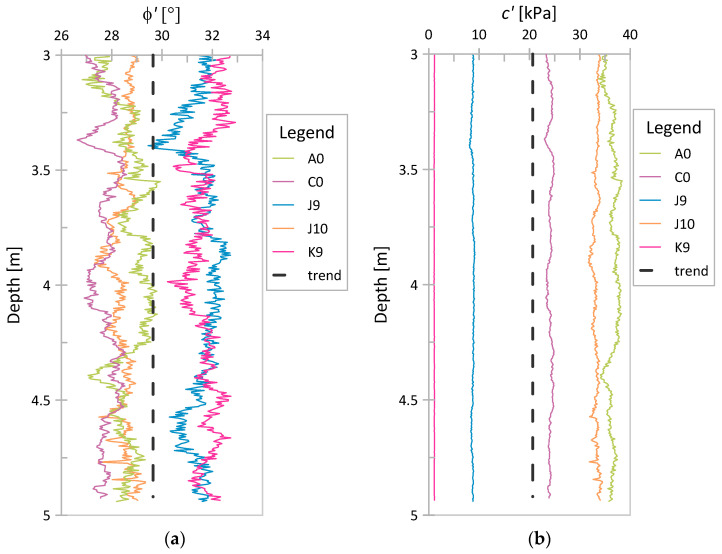
NTH results for Keswick clay: (**a**) *ϕ*′; (**b**) *c*′.

The obtained *ϕ*′ and *c*′ values for Świerzna clay and Keswick clay are presented in Figure 10 and Figure 11, respectively. As seen, the values in the figures are practically trendless. No trend was also observed when analyzing the COVs as a function of depth. For this reason, a single global coefficient of variation was determined for each parameter, referring to the values obtained at all considered points. The obtained values of the COV for individual cases are presented in Table 6. These results are discussed in the next section.

### 3.3. Shear Strength Parameters ϕ′ and c′ Determined by the Method of Equations

**Figure 12:**
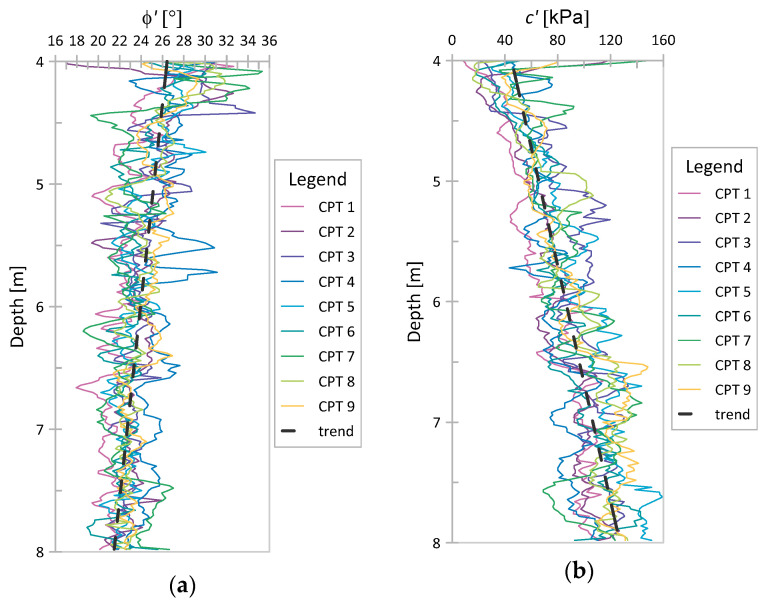
MoE results for Świerzna clay: (**a**) *ϕ*′; (**b**) *c*′.

**Figure 13:**
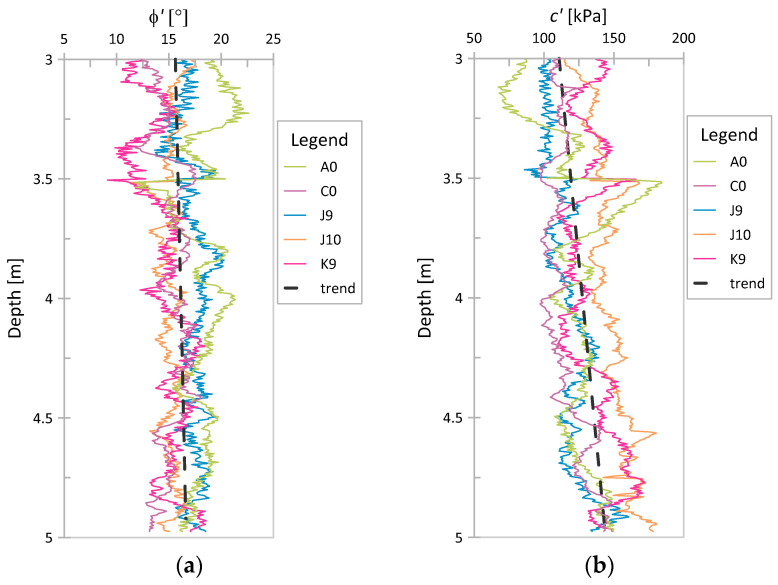
MoE results for Keswick clay: (**a**) *ϕ*′; (**b**) *c*′.

The values of *ϕ*′ and *c*′ were also investigated using the method of equations (MoE). To ensure that, the values of *q_c_* and *f_s_* obtained at each point of the considered CPT profiles were substituted into Equations (9)–(13). Since, in the case of Świerzna clay, the determined values of *u*_2_ in each considered point were a negligible percentage of the *q_t_* value, the calculation of *q_t_* and *q_E_* was omitted, as both of them were assumed to be equal to *q_c_*. A similar assumption was made for Keswick clay due to the lack of data on *u*_2_. The obtained values for *ϕ*′ and *c*′ for Świerzna clay and Keswick clay are presented in Figure 12 and Figure 13, respectively. The value of *c*′ presented here is several times higher than that derived from the NTH method in the previous section.

Moreover, both parameters obtained using the MoE are characterized by trends with depth (similar for both analyzed case studies). For *ϕ*′, it is a weak decreasing or increasing trend (depending on the case), and for *c*′, it is a strong increasing trend. Despite trends in parameter values, one global mean and one global COV value were determined (as before for the NTH method). These data are summarized in Table 7. Once again, the obtained results will be discussed in the next section.

### 3.4. Vertical SOF

**Figure 14:**
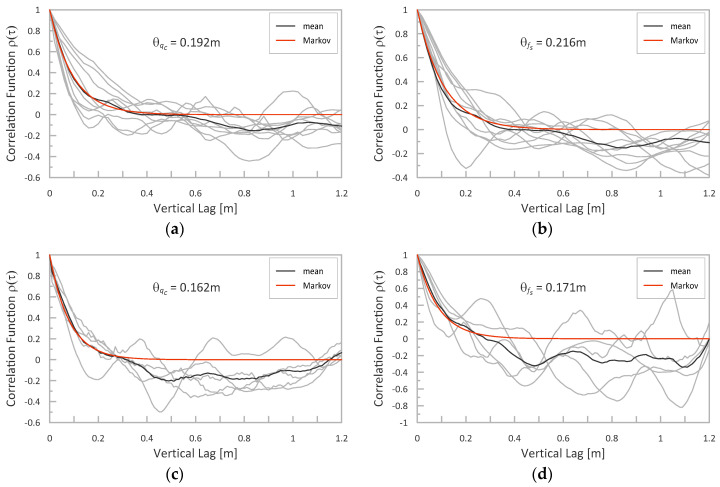
Vertical SOF obtained in analyzed case studies: (**a**) Świerzna clay-SOF for *q_c_*; (**b**) Świerzna clay-SOF for *f_s_*, (**c**) Keswick clay-SOF for *q_c_*; (**d**) Keswick clay-SOF for *fs*.

For both studies, the values of vertical SOFs for both the original *q_c_* and *f_s_* signals and the transformed values of *s_u_*, *ϕ*′, and *c*′ were determined. For the *q_c_* and *f_s_*, the obtained fits of the theoretical models to the experimental ACF averaged over all considered CPTs (marked with dark lines) together with the corresponding SOF values are presented in Figure 14. As seen, the Markov function corresponds well to the mean of the experimental data. Please note that the obtained value of the vertical scale in the case of Keswick clay is only slightly lower than that presented in work by Jaksa et al. [19]. These small differences may result from the selection of only a few CPT tests for this study, analyzing them only within the layer between 3 m and 5 m under the surface and using linear detrending (in work by Jaksa et al. [19], a quadratic trend for a larger depth range was used).

Similarly, the vertical SOF was also examined for the derived values of *s_u_*, *ϕ*′ an *c*′. All obtained values of SOF are reported in Table 8. These values will be discussed in the following section.

### 3.5. Cross-Correlation Coefficient ρ between c′and ϕ′

**Figure 15:**
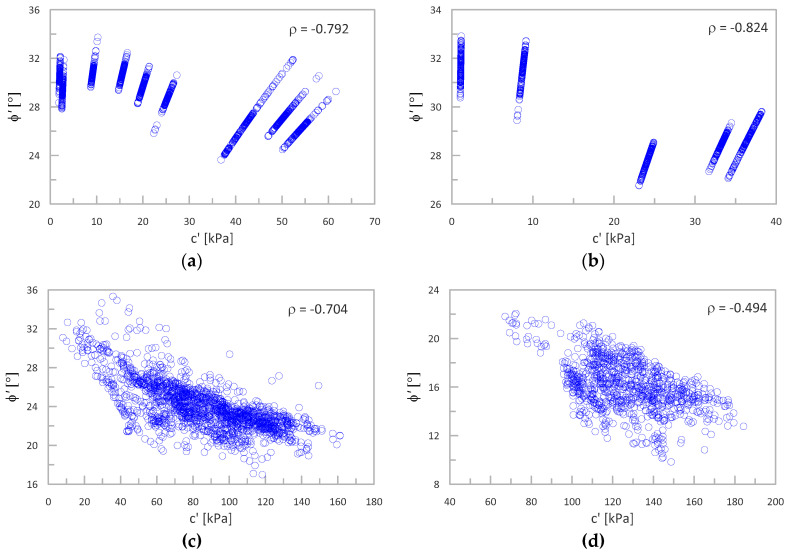
The value of the correlation coefficient *ρ*: (**a**) Świerzna clay (**b**) Keswick clay (the); (**c**) Świerzna clay (**d**) Keswick clay (the MoE).

Additionally, the Pearson’s cross-correlation coefficient *ρ* between the obtained parameters was analyzed. All obtained values of both parameters are presented in *c*′*-ϕ*′ space (for both analyzed cases) in Figure 15, together with the calculated value of the correlation coefficient. As seen, the obtained images using the NTH method (Figure 15a,b) are not typical: they consist of sets of points arranged in the form of straight lines. Each line corresponds to different CPT tests. To determine the *c*′ values using Equation (3) (for *a*′, which is constant for each profile), *ϕ*′ and *c*′ are fully correlated (*ρ* = 1). A more typical filling of the *c*′*-ϕ*′ space is observed in the case of MoE (Figure 15c,d). In both cases, the resulting global values of the coefficient *ρ* in the whole *c*′ *ϕ*′ space are negative and acceptable (similar to literature data). These results will also be discussed in the next section.

## 4. Discussion of Results

As mentioned in the introduction section, this paper aims to test the possibility of identifying random fields of soil strength parameters based only on transformed CPT signals. In this section, the results presented in the previous section are discussed in this context.

### 4.1. Variability in the Undrained Shear Strength s_u_

The results presented in Section 3 show that *s_u_* values are well correlated with *q_c_* registration, which is mainly evidenced by obtaining similar *s_u_* values and trends based on laboratory tests and selected CPT tests in Keswick clay. Although the obtained trends have different coefficients, the linear functions in the analyzed layer are almost identical. In the case of Świerzna clay, the value of the direction coefficient of the trend function obtained based on the CPT results is similar to that of Keswick clay, which may indicate similar behavior of this soil.

Comparing the COV values for Keswick clay derived based on CPT results as a function of depth, with the COV values calculated for the same soil for a few different depths based on laboratory tests (Table 2), it can be concluded that the values from CPT in the considered layer are generally lower than those based on laboratory tests. However, the COVs for samples taken slightly below the considered layer (at a depth of 7 m) are of the same order as those received based on CPT. The variability in the results of laboratory tests may be influenced by many additional factors related to collecting the samples or test methodology. In addition, although CPT tests lying at a certain distance from each other (two groups at a distance of approximately 70 m) have been selected for analysis, the authors have no information on the area from which the samples for laboratory tests were taken for Keswick clay (it is quite likely that they were taken from a larger area). In this respect, the slightly lower variability of field test results is not surprising, but it seems justified. The fluctuation scale, mean value, trend, and COV identified based on the CPT results form a complete set of information needed to identify a weakly stationary random field. The occurrence of a strong trend in the results indicates that this trend should be included in the analysis. Thus, for the final modeling, a trend should be added to SRF (identified based on detrended values). This method of *s_u_* modeling has been already used in some works (e.g., [51]).

It should be noted that good agreement between trends based on laboratory and CPT results was obtained for the results of selected CPTs (with *q_c_* values rising with depth) and analyzing them within a homogeneous soil layer (its homogeneity was decided based on similar CPT registrations). The authors believe that only under such conditions is it possible to reconstruct the trend and make a reasonable estimation of the coefficient of variation of the *s_u_* parameter (c.f., e.g., [16]).

### 4.2. Variability in ϕ′ and c′ Based on the NTH Method and MoE

In terms of determining values of *ϕ*′ and *c*′, much more reliable values of these parameters for the analyzed soil were obtained based on the NTH method. Comparing Figure 13b and Figure 14b with Figure 9, one can obtain the impression that the values of *c*′ (for MoE) are closer to *s_u_* and do not correspond to the effective values of cohesion obtained in both triaxial and direct shear tests (the obtained values are significantly larger than the typical values of *c*′ from laboratory tests). Thus, although the concept of the method of equations is interesting and tempting, to provide reliable values of effective parameters, the method needs to be further developed.

The graphs of strength parameters obtained using the NTH method for both analyzed case studies (Figure 11 and Figure 12) show that both *ϕ*′ and *c*′ are practically trendless. The results of laboratory tests investigate the values of effective parameters at different depths in clays (e.g., [39]). This observation also confirms the correctness of the assumption of stationarity in the construction of random fields of effective strength parameters.

The variability in soil parameters resulting from the NTH method (Table 6) fits quite well with the values obtained from laboratory tests for these parameters (Table 3). Similarly, as in laboratory tests, the variability in *c*′ is greater than the variability in *ϕ*′. The obtained COV values are also within the ranges given for each parameter. Thus, referring to the parameters of SRF modeling *c*′ and *ϕ*′, it seems that the estimation of COV values can be obtained based on the interpretation of the CPT test.

### 4.3. Vertical Scale of Fluctuation for Original and Transformed Signals

As seen in Table 8, the vertical fluctuation scales for *s_u_* as well as *ϕ*′ and *c*′ obtained using the NTH method are similar to those determined for *q_c_* and *f_s_*. The only case of significant differences in SOF values is the scale determined for parameter *ϕ*′ obtained from the MoE. Its value is 15–25% lower than the SOF obtained in all other cases. As this is expected, since some of the transformations are linear or almost linear, it generally confirms that the scale of fluctuation for strength parameters can be assumed identically as the one identified for the original signal, which is a common practice.

### 4.4. Cross-Correlation Coefficient ρ

The presented results show that based on both methods, a reliable value of the correlation coefficient ρ is obtained. The NTH method gives a specific image of results in the *c*′-*ϕ*′ space related to the procedure. However, the final values obtained for the whole space are within the range of literature data.

## 5. Conclusions

In this work, the procedure for identifying the uncertainty in strength parameters for cohesive soils using only CPT results and random fields was shown. Existing methods of interpretation of CPT tests regarding strength parameters of such soils, i.e., *s_u_* or *c*′ and *ϕ*′, were used to transform the measurements of CPT probe and determine the mean values, trends (if they exist), COV, and SOF for these parameters. The determination of the cross-correlation coefficient between the *c*′ and *ϕ*′ parameters was also investigated. For comparison purposes, the same procedure was used to analyze the CPT results obtained in two different case studies.

Based on the presented results, the following conclusions can be drawn:

- The uncertainty of the model parameters is an essential issue, which once identified allows for managing specific resources. In numerical modeling of geotechnical structures, the problem can refer to the uncertainty in shear strength parameters. Understanding this uncertainty enables one to manage the risk of failure by designing the structure for the specific failure probability. While in typical numerical studies, the strength parameters of soils are modeled as constant, using SRF to describe this uncertainty is the approach with rising interest.
- When stationary random fields are used to model soil strength parameters, data from two different sources are typically used for their identification; the scale of fluctuation is assessed based on CPT and point statistics based on laboratory tests, which are often limited in number. More sophisticated statistical modeling approaches are based on large databases of both filed and laboratory test results, which are rarely available and associated with high costs. The proposed procedure allows for identifying the parameters of SRF for cohesive soil in the case of insufficient information regarding laboratory test results, based only on CPT measurements. Despite the relatively simple approach of analyzing the directly transformed CPT signal, the resulting measures of variability, which allow identifying the random SRF, appear to agree very well with the literature data.
- The presented procedure applied to the *s_u_* parameter in Keswick clay (for which both laboratory and field test results were available) predicted a similar trend of *s_u_* to that obtained based on laboratory tests. Although the COV value obtained based on those CPTs appeared to be slightly less than ten from laboratory tests, it remains in a reasonable range. Moreover, the COV values for the *ϕ*′ and *c*′ parameters as well as the cross-correlation coefficient ρ for these parameters (obtained using the NTH method) fall within the range of typical values obtained in laboratory investigations.
- The lack of strong trends of *c*′ and *ϕ*′ resulting from the NTH method confirmed by laboratory test results proves that modeling these parameters by stationary random fields (which is a common practice) is correct. However, that is not the case for *s_u_*. The strong trend obtained for that parameter should be accounted for in its random field representation.
- The performed analysis shows that the fluctuation scale determined for *q_c_* or *f_s_* does not change when the obtained values are transformed to *s_u_*, or *ϕ*′ and *c*′ (particularly in the case of the NTH method). As the transformations used are practically linear, it is not surprising. These results, however, are in line with the commonly used assumption that the fluctuation scales for different parameters should be equal.
- The value of *c*′ obtained using the method of equations significantly differs from the values obtained from the NTH method. It seems that the value of cohesion obtained with this method does not represent the effective value of cohesion *c*′, and the method probably needs to be further developed. The concept of the method is, however, very interesting and seems worthy of further investigation.

The proposed approach seems promising. It seems that in the absence of large databases, random fields of strength parameters can be identified using only the transformed CPT signals. Such identification allows for stochastic modeling of geoengineering structures located in the investigated soil. However, before the actual application, the proposed method should be verified based on dedicated field and laboratory tests in specific soils. This verification is currently the subject of further investigation by the authors.

## Figures and Tables

**Table 1:** Basic characteristics of Świerzna and Keswick clay.

Soil	Unit Weight [kN/m^3^]	IP [%]	OCR [-]
Świerzna clay	22.02 ± 0.39 (from laboratory test)	19.2 ± 2.3 (from laboratory test)	6.77 ± 0.61 (from CPT korelation)
Keswick clay	18 (Jaksa PhD [44])	25–88 (Jaksa PhD [44])	7.64 ± 0.33 (from CPT korelation)

**Table 2:** Variability of Keswick clay according to Appendix C to [43].

Soil	Triaxial Test Type	No. of Samples (up to 10 m Depth)	Trends Equation [kPa]	Depth *z* [m]	COV [-]
Overconsolidated clay (Keswick clay)	UU and CU	75 UU and 12 CU	*s_u_* = 16.202 *z* + 55.128	3.2	0.33
				4.7	0.19
				7.7	0.13

**Table 3:** Typical values of COV and *ρ* for effective shearing parameters of cohesive soils.

Reference	Soil Type		Test Type	No. of Samples	COV for *ϕ*′	COV for *c*′	Parsons Coeff. *Ρ*
Sevaldson [47]	Lightly overconsolidated clay (Lodalen landslide)		Triaxial CD	10	0.060	0.210	−0.070
Wolff et al. [48]	Bois Brule Levee embankment and foundation clay		Triaxial CD	9	0.099–0.0165	1.280–1.310	−0.388–−0.694
Hata et al. [49]	The cohesive soil-forming subsoil of Airports in Japan	APIII	Triaxial	14CU	0.192	1.068	-
		APIX		14CU	0.105	0.880	-
		APX		10CD	0.115	0.958	−0.557
Di Matteo et al. [50]	Silty clay		Direct shear	16	0.030	0.210	−0.925

**Table 4:** Trends in COV values for *s_u_* obtained based on CPT results (*z* is depth).

Soil	Trends Equation [kPa]	Range of COV Values [-]	Mean COV [-]
Swierzna clay	*s_u_* = 19.407 *z* + 19.539	0.068–0.425	0.152
Keswick clay	*s_u_* = 22.495 *z* + 30.620	0.038–0.186	0.105

**Table 5:** *a*′ values for the considered sections of CPT profiles.

	Global Value (All CPTs)	CPT1	CPT2	CPT3	CPT4	CPT5	CPT6	CPT7	CPT8	CPT9
Świerzna Clay *a*′ [kPa]	44.19	4.7	46.1	110.0	98.2	15.3	3.4	84.2	35.1	26.2
Keswick clay *a*′ [kPa]	33.05	CPTA0		CPTC0		CPTJ10		CPTJ9		CPTK9
		66.7		45.8		14.2		61.3		1.8

**Table 6:** Variability measures of parameters according to the NTH method.

	*ϕ*′		*c*′	
	Mean [°]	COV [-]	Mean [°]	COV [-]
Świerzna clay	28.88	0.066	24.60	0.767
Keswick clay	29.64	0.058	20.65	0.663

**Table 7:** Variability measures of parameters according to the MoE.

	*ϕ*′		*c*′	
	Mean [°]	COV [-]	Mean [°]	COV [-]
Świerzna clay	23.95	0.103	85.90	0.339
Keswick clay	16.12	0.127	127.16	0.161

**Table 8:** The scale of fluctuation values for each method of estimation of strength parameters.

Case Study	The Scale of Fluctuation (m)						
	Directly Measured by CPT		Undrained	Drained, MoE		Drained, NTH Method	
	*q_c_*	*f_s_*	*s_u_*	*ϕ*′	*c*′	*ϕ*′	*c*′
Świerzna clay	0.192	0.216	0.184	0.183	0.214	0.189	0.188
Keswick clay	0.162	0.171	0.166	0.121	0.166	0.164	0.164
